# Retinal and coronary imaging in type 1 diabetes: links between retinopathy and subclinical cardiovascular disease

**DOI:** 10.3389/fcvm.2026.1963643

**Published:** 2026-10-07

**Authors:** Lucie Lyskova, David Karasek

**Affiliations:** 3rd Department of Internal Medicine – Nephrology, Rheumatology and Endocrinology, University Hospital Olomouc and Faculty of Medicine and Dentistry, Palacký University Olomouc, Olomouc, Czech Republic

**Keywords:** cardiovascular disease, coronary artery calcium, coronary computed tomography angiography, diabetic retinopathy, oculomics, retinal imaging, subclinical atherosclerosis, type 1 diabetes mellitus

## Abstract

Cardiovascular disease (CVD) remains the leading cause of premature morbidity and mortality in individuals with type 1 diabetes mellitus (T1DM), despite advances in glycemic management and cardiovascular prevention. Conventional cardiovascular risk assessment may underestimate risk in T1DM, particularly among younger asymptomatic individuals, highlighting the need for markers of early vascular injury. Diabetic retinopathy (DR), a microvascular complication of T1DM, is increasingly recognized as a marker of systemic vascular dysfunction rather than an isolated ocular disorder. Non-invasive retinal visualization may provide insight into vascular and atherosclerotic disease. This mini-review summarizes evidence linking DR with subclinical coronary artery disease (CAD) and cardiovascular outcomes in T1DM, focusing on coronary artery calcium (CAC) and coronary computed tomography angiography (CCTA). Observational and longitudinal studies consistently link DR severity with CAC burden, coronary plaque burden, silent CAD, and cardiovascular outcomes, independent of traditional risk factors. Albuminuria and diabetic kidney disease may provide complementary evidence of a broader microvascular phenotype associated with cardiovascular risk. Advances in retinal imaging, including optical coherence tomography angiography and artificial intelligence-based oculomics, support the role of retinal biomarkers in cardiovascular risk prediction. CAC scoring remains valuable for detecting subclinical coronary atherosclerosis, while CCTA characterizes calcified and non-calcified plaque. Emerging evidence suggests that integrating retinal and microvascular markers with coronary imaging may improve identification of asymptomatic individuals at increased cardiovascular risk and support intervention. Although data are observational and prospective validation is required, multimodal strategies combining clinical risk factors, retinal biomarkers, albuminuria, CAC, and CCTA may enhance cardiovascular risk stratification in T1DM.

## Introduction

Type 1 diabetes mellitus (T1DM) is a chronic autoimmune disorder characterized by absolute insulin deficiency and lifelong exposure to hyperglycemia. Despite substantial advances in insulin replacement therapy, continuous glucose monitoring, and cardiovascular prevention strategies, individuals with T1DM continue to face a markedly elevated risk of cardiovascular disease (CVD), which remains the leading cause of premature morbidity and mortality in this population (1, 2). Compared with the general population, patients with T1DM have a significantly higher incidence of coronary artery disease (CAD), heart failure, stroke, and peripheral arterial disease, with cardiovascular complications frequently occurring at younger ages (1, 3). Although improvements in glycemic management and comprehensive risk-factor control have contributed to declining cardiovascular mortality rates, considerable residual cardiovascular risk persists even among individuals achieving recommended glycemic targets, suggesting that factors beyond chronic hyperglycemia contribute to ongoing vascular injury (1, 3).

The increased cardiovascular burden in T1DM reflects the early onset and long duration of the disease, resulting in prolonged exposure to metabolic and vascular stressors throughout life. However, current cardiovascular risk prediction models are largely derived from populations with type 2 diabetes mellitus (T2DM) and may inadequately capture the unique clinical and pathophysiological features of T1DM, particularly in younger, asymptomatic individuals (4, 5). The pathogenesis of CVD in T1DM is multifactorial, involving chronic hyperglycemia, endothelial dysfunction, oxidative stress, low-grade inflammation, dyslipidemia, glycemic variability, recurrent hypoglycemia, diabetic kidney disease, and immune-mediated vascular injury, all contributing to accelerated atherosclerosis and both microvascular and macrovascular complications (1, 6). Importantly, unlike T2DM, cardiovascular disease in T1DM often develops in the absence of obesity or metabolic syndrome, suggesting that traditional cardiovascular risk factors alone do not fully explain the observed excess risk (1, 4). Large registry-based studies have identified glycated hemoglobin (HbA1c), albuminuria, impaired renal function, hypertension, and diabetes duration as major predictors of cardiovascular morbidity and mortality in T1DM (3). Nevertheless, cardiovascular events frequently occur even in individuals without advanced conventional risk factors, suggesting that additional disease-specific mechanisms contribute to accelerated vascular injury and underscoring the need for improved cardiovascular risk stratification (1, 5).

Long-term follow-up of participants in the Diabetes Control and Complications Trial (DCCT) and its observational extension, the Epidemiology of Diabetes Interventions and Complications (EDIC) study, has demonstrated that diabetic retinopathy, diabetic kidney disease, and diabetic neuropathy are strongly associated with subsequent cardiovascular outcomes, highlighting the close interplay between microvascular and macrovascular complications in T1DM (7, 8). The coexistence of retinal and renal microvascular abnormalities may therefore provide complementary information on systemic vascular injury (8). Among these complications, diabetic retinopathy (DR) is one of the most prevalent and well-characterized manifestations of microvascular damage. Increasing evidence suggests that DR should be viewed not merely as an ocular complication but as a marker of systemic vascular injury. Because the retinal microvasculature can be directly and non-invasively visualized, retinal abnormalities may provide a unique window into generalized endothelial dysfunction, microvascular impairment, and early vascular damage throughout the body (9). Recent advances in retinal imaging technologies, including optical coherence tomography angiography (OCTA), adaptive optics imaging, and artificial intelligence (AI)-based approaches, have further expanded the clinical potential of retinal biomarkers for cardiovascular risk assessment (10–12) In particular, the emerging field of oculomics integrates high-resolution retinal imaging with machine-learning approaches to identify retinal characteristics associated with cardiovascular and other systemic diseases. By extracting subtle vascular and structural features beyond conventional clinical assessment, oculomics offers a scalable, non-invasive, and potentially cost-effective strategy for the early detection of individuals at increased cardiovascular risk (10, 11).

Coronary artery calcium (CAC) scoring is a well-established, non-invasive imaging modality for detecting subclinical coronary atherosclerosis and refining cardiovascular risk stratification in asymptomatic individuals (13, 14). In T1DM, increased CAC burden may be present even in the absence of clinically overt CAD, reflecting underlying atherosclerotic changes. However, because CAC scoring quantifies only calcified plaque, it may underestimate the extent of early coronary atherosclerosis, particularly in younger individuals in whom non-calcified plaques predominate. In this setting, coronary computed tomography angiography (CCTA) provides a more comprehensive assessment of coronary plaque burden and composition, by detecting both calcified and non-calcified lesions (15, 16). Recent CCTA studies have demonstrated that asymptomatic individuals with T1DM may harbor significant coronary atherosclerosis despite the absence of clinically manifest cardiovascular disease, with younger patients often exhibiting predominantly non-calcified plaque phenotypes not adequately captured by CAC assessment alone (16, 17).

In this population, the clinical relevance of detecting subclinical coronary abnormalities lies primarily in identifying early systemic vascular injury and atherosclerotic burden rather than established clinical CAD. This distinction is particularly relevant in young adults, in whom clinically manifest coronary disease is uncommon but early vascular abnormalities may already be detectable.

This mini-review summarizes current evidence regarding the association between diabetic retinopathy and cardiovascular disease in T1DM, with particular emphasis on coronary artery calcium, subclinical coronary atherosclerosis, and the retinal imaging for early cardiovascular risk prediction.

## Diabetic retinopathy as a marker of systemic cardiovascular disease

Beyond its ophthalmological consequences, diabetic retinopathy (DR) is increasingly recognized as a marker of systemic vascular disease and adverse cardiovascular outcomes in individuals with T1DM (9, 18). Progression of DR is characterized by retinal ischemia, endothelial dysfunction, and microvascular remodeling, mechanisms that overlap with those underlying systemic atherosclerosis. Typical fundus features of non-proliferative and proliferative DR are presented in Figure 1. This biological overlap supports the concept that retinal microvascular abnormalities reflect underlying coronary atherosclerosis (6, 9).

**Figure 1:**
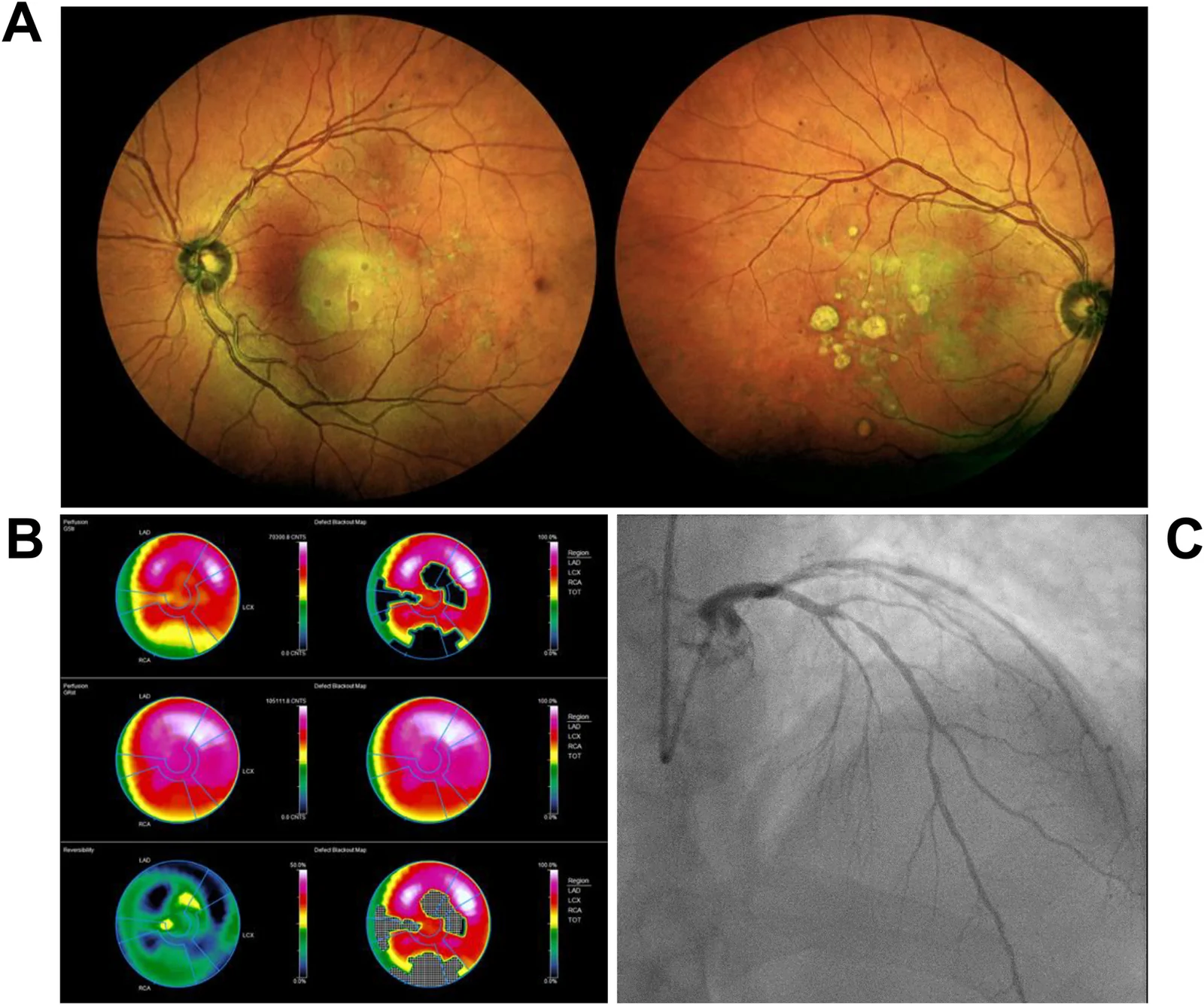
Multimodal assessment of retinal and subclinical coronary disease in a patient with T1DM. Diabetic retinopathy screening: Non-proliferative diabetic retinopathy in the right eye and proliferative diabetic retinopathy in the left eye **(A)**. Myocardial stress perfusion scintigraphy: severe reversible ischemia **(B)**. Coronary angiography: tandem 80% stenosis in the proximal and middle parts of the left anterior descending artery and a 60% distal lesion in the left circumflex artery **(C)**.

Epidemiological evidence consistently demonstrates that DR is associated with increased cardiovascular morbidity and mortality independently of conventional cardiovascular risk factors. A meta-analysis including more than 11,000 individuals with diabetes reported significantly higher cardiovascular mortality among patients with DR, while severe DR was associated with an approximately twofold increase in cardiovascular mortality risk (19). Similarly, longitudinal studies in T1DM demonstrated strong associations between severe DR and coronary heart disease, peripheral arterial disease, and major adverse cardiovascular events (20).

Longitudinal evidence further strengthens the role of DR as a cardiovascular risk marker in T1DM. In a nationwide matched case–cohort study, increasing DR severity was associated with progressively higher risk of incident cardiovascular disease, while pre-existing cardiovascular disease independently predicted future progression of DR (18).

Emerging retinal imaging techniques may identify vascular abnormalities before advanced clinical DR develops. Adaptive optics retinal imaging using the rtx1 camera demonstrated increased retinal arteriolar wall thickness and wall-to-lumen ratio in children with T1DM compared with healthy controls, suggesting that subtle microvascular abnormalities may be detectable at an early stage of disease (12). Recent advances in retinal imaging, particularly OCTA, have further supported the association between retinal microvascular abnormalities and subclinical coronary atherosclerosis (21).

The relationship between retinal and renal microvascular disease provides additional support for a broader systemic microvascular phenotype. In the DCCT/EDIC cohort, advanced albuminuria and the coexistence of retinal and kidney disease were associated with subsequent cardiovascular disease, suggesting that retinal and renal microvascular abnormalities may identify overlapping patterns of systemic vascular injury (8). More recently, the LIFE-T1D model incorporated both albuminuria and retinopathy among predictors of cardiovascular risk in individuals with T1DM, further supporting their complementary role in cardiovascular risk assessment (22).

## Coronary artery calcium and coronary CTA in T1DM

Subclinical CAD is increasingly recognized as an important component of cardiovascular risk in individuals with T1DM, particularly in those with long disease duration and microvascular complications (13, 14). Because clinically overt cardiovascular disease may develop only after years of silent atherosclerotic progression, imaging methods capable of detecting early coronary disease have become increasingly relevant in asymptomatic individuals with T1DM.

Coronary artery calcium (CAC) scoring is strongly associated with future cardiovascular events and mortality in patients with diabetes (5, 13). In T1DM, studies demonstrated substantial heterogeneity in CAC burden, ranging from absent coronary calcification to extensive coronary atherosclerosis despite the absence of cardiovascular symptoms (15).

Consistent with these observations, progression of CAC in individuals with long-standing T1DM was associated with the presence and severity of DR, suggesting that retinal microvascular disease may parallel progression of coronary atherosclerosis (23).

Despite its prognostic value, CAC scoring may underestimate total coronary plaque burden, particularly in younger individuals with predominantly non-calcified plaques (16). Coronary computed tomography angiography (CCTA) therefore provides complementary information through direct assessment of coronary stenosis, plaque burden, plaque composition, and high-risk plaque features (24, 25). Recent CCTA studies demonstrated that asymptomatic individuals with T1DM may exhibit substantial non-calcified coronary plaque burden despite relatively low CAC scores (16).

Recent advances in artificial intelligence (AI)-assisted coronary imaging have further expanded the ability to characterize coronary atherosclerosis beyond conventional CAC assessment. These technologies enable automated and highly detailed quantification of total plaque burden, plaque composition, and high-risk plaque characteristics (16, 26).

CAC scoring and CCTA provide complementary approaches for detecting coronary atherosclerosis in individuals with T1DM. Association Between Diabetic Retinopathy and Subclinical Coronary Artery Disease in Type 1 Diabetes Mellitus.

Growing evidence supports a close association between DR and subclinical CAD in individuals with T1DM. Across observational, longitudinal, and imaging studies, retinal microvascular abnormalities consistently correlate with coronary calcification, coronary plaque burden, and subclinical CAD independently of conventional cardiovascular risk factors (16, 23, 27, 28). The principal clinical studies supporting these associations are summarized in Table 1.

**Table 1:** Key studies investigating the association between diabetic retinopathy and subclinical coronary artery disease in diabetes.

Reference	Study population	Assessment	Key finding
DCCT/EDIC Research Group	1,441 participants with T1DM (DCCT/EDIC cohort)	Clinical CVD outcomes	Microvascular complications were associated with future CVD.
Pongrac Barlovic et al. (20)	1,683 adults with long-standing T1DM (>30 years)	Clinical CVD outcomes	Severe DR independently predicted cardiovascular events.
Kawasaki et al. (MESA) (27)	927 participants with diabetes from the MESA cohort	CAC	Vision-threatening DR was idependently associated with greater CAC burden.
Simó et al. (PRECISED) (28)	200 patients with T2DM without clinical CVD and 60 age-matched controls	CAC + CCTA	Increasing DR severity was associated with silent CAD and greater plaque burden.
Yang et al. (21)	1,286 asymptomatic adults at increased cardiovascular risk	OCTA + CCTA	Reduced retinal vascular density was associated with subclinical coronary atherosclerosis.
Mabala et al. (18)	16,547 adults with T1DM attending nationwide diabetic eye screening	Incident CVD	Increasing DR severity predicted incident CVD; pre-existing CVD predicted DR progression.

Among the earliest studies, the Multi-Ethnic Study of Atherosclerosis (MESA) demonstrated that vision-threatening DR was independently associated with greater CAC burden and subclinical cardiovascular disease after adjustment for traditional cardiovascular risk factors, suggesting that retinal microvascular pathology provides additional information beyond conventional cardiovascular risk assessment (27).

Subsequent studies in individuals with long-standing T1DM further strengthened this association. Progression of CAC was independently associated with DR severity and other microvascular complications (23), suggesting that retinal microvascular disease may parallel progression of coronary calcification.

More detailed characterization of this relationship was provided by the PRECISED study. In asymptomatic individuals with diabetes, DR was independently associated with elevated CAC scores, silent CAD, and coronary stenosis detected by coronary computed tomography angiography (CCTA) (28). Individuals with moderate-to-severe DR exhibited a substantially higher prevalence of coronary atherosclerosis than individuals with mild or no retinopathy, and these associations remained significant after adjustment for HbA1c, diabetes duration, renal function, and conventional cardiovascular risk factors.

Further evidence has been provided by advanced coronary imaging. AI-assisted CCTA demonstrated that asymptomatic individuals with T1DM may exhibit substantial non-calcified coronary plaque burden despite relatively low CAC scores (16). Similarly, OCTA studies linked reduced retinal vascular density with CAC burden, obstructive CAD, and overall coronary plaque burden assessed by CCTA (21). Together, these findings further support the role of retinal microvascular abnormalities as markers of subclinical CAD.

Longitudinal evidence further suggests a bidirectional relationship between DR and cardiovascular disease progression. In a nationwide matched case–cohort study, increasing DR severity was associated with progressively higher cardiovascular risk, while pre-existing cardiovascular disease independently predicted future progression of DR (18). This reciprocal relationship suggests that retinal and coronary vascular disease may represent interconnected manifestations of generalized vascular pathology.

Current evidence consistently supports an independent association between DR and subclinical CAD in individuals with T1DM. These findings suggest that retinal imaging may complement coronary imaging to aid in the identification of asymptomatic individuals at increased cardiovascular risk – see Figure 1.

## Discussion and future perspectives

Current evidence suggests that diabetic retinopathy (DR) may provide clinically relevant information regarding cardiovascular risk in individuals with type 1 diabetes mellitus (T1DM) beyond conventional cardiovascular risk stratification models. Because DR is routinely evaluated during standard ophthalmological screening, retinal findings may represent an accessible and non-invasive marker for identifying individuals at increased risk of occult CAD (18, 19, 28).

One of the most important clinical implications of the observed association between DR and subclinical CAD is the potential refinement of cardiovascular risk stratification in T1DM. In this context, the presence of moderate-to-severe DR may identify patients with an increased likelihood of subclinical CAD who could benefit from intensified preventive management and additional cardiovascular evaluation (23, 28).

The combination of retinal assessment with coronary imaging may be particularly valuable in asymptomatic individuals without clinically manifest cardiovascular disease. Rather than replacing coronary imaging, retinal findings may help identify patients who are most likely to benefit from further cardiovascular evaluation when conventional cardiovascular risk stratification is insufficient. This multimodal approach may facilitate earlier detection of subclinical CAD while potentially avoiding unnecessary imaging in lower-risk individuals. As coronary imaging techniques continue to evolve, integration of retinal assessment with contemporary coronary imaging may further improve the identification of early coronary atherosclerosis.

Beyond conventional fundus examination, emerging retinal imaging technologies, including OCTA, adaptive optics, and AI-assisted retinal image analysis, may further expand the **potential** role of retinal biomarkers in cardiovascular medicine.

Despite promising findings, several limitations of the currently available evidence should be acknowledged. Most studies evaluating the relationship between DR and CAD were observational and included relatively limited numbers of participants (16, 23, 28). In addition, heterogeneity in DR classification, imaging protocols, and cardiovascular endpoints complicates direct comparison between studies. Evidence directly integrating retinal biomarkers, albuminuria, and coronary imaging remains limited, and therefore the incremental value of combining these markers cannot yet be established. Importantly, it remains uncertain whether the incorporation of retinal biomarkers into cardiovascular assessment algorithms improves long-term clinical outcomes in T1DM.

Future research should focus on studies integrating retinal biomarkers with coronary imaging and established clinical risk factors. Standardization of retinal imaging biomarkers and validation of AI-assisted risk prediction models will be important steps toward future clinical implementation.

## Conclusion

Collectively, available evidence suggests that diabetic retinopathy (DR) should no longer be regarded solely as an isolated microvascular ocular complication in individuals with type 1 diabetes mellitus (T1DM). Rather, retinal microvascular abnormalities reflect generalized vascular pathology and are consistently associated with coronary artery calcium burden, subclinical CAD, and adverse coronary plaque characteristics. Together with albuminuria and diabetic kidney disease, these findings support the concept of an interconnected systemic microvascular phenotype in T1DM.

These findings suggest that conventional cardiovascular risk assessment may underestimate subclinical coronary atherosclerosis in asymptomatic individuals with long-standing T1DM. Integrating retinal assessment with contemporary coronary imaging may therefore improve identification of individuals at increased cardiovascular risk before clinically manifest cardiovascular disease develops.

Although current evidence remains predominantly observational, retinal biomarkers represent a promising and clinically accessible component of future multimodal cardiovascular risk stratification. Importantly, current risk prediction approaches do not yet provide a standardized framework integrating clinical risk factors, retinal biomarkers, and coronary imaging. Future prospective studies should therefore determine whether such multimodal strategies improve personalized cardiovascular prevention and long-term cardiovascular outcomes.
